# Proteolysis, NaOH and Ultrasound-Enhanced Extraction of Anticoagulant and Antioxidant Sulfated Polysaccharides from the Edible Seaweed, *Gracilaria birdiae*

**DOI:** 10.3390/molecules191118511

**Published:** 2014-11-13

**Authors:** Gabriel Pereira Fidelis, Rafael Barros Gomes Camara, Moacir Fernandes Queiroz, Mariana Santana Santos Pereira Costa, Pablo Castro Santos, Hugo Alexandre Oliveira Rocha, Leandro Silva Costa

**Affiliations:** 1 Laboratory of Biotechnology of Natural Polymers (BIOPOL), Department of Biochemistry, Federal University of Rio Grande do Norte (UFRN), Natal-RN 59078-970, Brazil; E-Mails: gabrielfideliss@gmail.com (G.P.F.); rafael_bgc@yahoo.com.br (R.B.G.C.); moacirfqn@gmail.com (M.F.Q.); marispc_bio@yahoo.com.br (M.S.S.P.C.); pabllocastro@hotmail.com (P.C.S.); hugo@cb.ufrn.br (H.A.O.R.); 2 Federal Institute of Education, Science and Technology of Rio Grande do Norte (IFRN), Santa Cruz-RN 59200-000, Brazil; 3 Department of Odontology, University of State of Rio Grande do Norte (UERN), Caicó-RN 59300-000, Brazil

**Keywords:** aPTT assay, total capacity antioxidant, sonication, proteolysis, red seaweed

## Abstract

The sulfated polysaccharides (SP) from the edible red seaweed, *Gracilaria birdiae*, were obtained using five different extraction conditions: *Gracilaria* birdiae 1 (GB1)-water; GB1s-water/sonication; GB1sp-water/sonication/proteolysis; GB2s-NaOH/sonication; and GB2sp-NaOH/sonication/proteolysis. The yield (g) increased in the following order: GB2sp > GB1sp > GB2s > GB1s > GB1. However, the amount of SP extracted increased in a different way: GB2sp > GB1 > GB1sp > GB1s > GB2s. Infrared and electrophoresis analysis showed that all conditions extracted the same SP. In addition, monosaccharide composition showed that ultrasound promotes the extraction of polysaccharides other than SP. In the prothrombin time (PT) test, which evaluates the extrinsic coagulation pathway, none of the samples showed anticoagulant activity. While in the activated partial thromboplastin time (aPTT) test, which evaluates the intrinsic coagulation pathway, all samples showed anticoagulant activity, except GB2s. The aPTT activity decreased in the order of GB1sp > GB2sp > GB1 > GB1s > GB2s. The total capacity antioxidant (TCA) of the SP was also affected by extraction condition, since GB2s and GB1 showed lower activity in comparison to the other conditions. In conclusion, the conditions of SP extraction influence their biological activities and chemical composition. The data revealed that NaOH/sonication/proteolysis was the best condition to extract anticoagulant and antioxidant SPs from *Gracilaria birdiae*.

## 1. Introduction

Seaweeds are a rich source of various compounds widely used in medicine, pharmaceuticals, cosmetics and food industries. Among these compounds, sulfated polysaccharides (SP) extracted from seaweeds occupy a special place, because they exhibit a wide range of activities when tested in different biological systems displaying anti-adhesive, antioxidant, antiviral, antitumor, antiulcerogenic, antithrombotic and anticoagulant properties [1,2].

The most extensive studies have been conducted on the anticoagulant activities of several algal SP [3,4,5], and more recently, the antioxidant and antiproliferative activities have been widely studied [1,6].

*Gracilaria birdiae*, a red seaweed distributed around the tropical region of Brazil, comes from an economically important genus named *Gracilaria*, which is used to obtain agar [7]. *Gracilaria* spp. is found on a large scale in the northeast of Brazil; however, population growth and increasing urbanization have resulted in the depletion of natural beds. In order to minimize this devastation, communities are cultivating seaweed both for commercial purposes and for the protection of these natural areas [8]. In Brazil, *G. birdiae* has been cultivated under field conditions in several locations, such as Rio do Fogo Beach (Rio Grande do Norte state), which produces biomass ranging from 900 to 3l,537 g/m^2^ [9]. In Rio do Fogo Beach, this alga is also used to feed native people.

In addition to the benefits already cited, SP-rich extracts from *G. birdiae* have been shown to prevent naproxen-induced gastrointestinal damage in rats [10], ameliorate trinitrobenzenesulfonic acid-induced colitis in rats [11] and present antioxidant [12] and anti-inflammatory [13] properties.

The SP algal biological activity usually depends on molecular size, type of sugar and sulfated contents, as well as the type of linkage, molecular geometry and sulfated position [1]. These structural variations determine not only the SP activity, but also their mechanisms of action [14]. In addition, several researchers have proposed that the structure of the different polysaccharides may vary according to the method of extraction [15,16,17].

There is no standard method to extract sulfated polysaccharides from seaweeds. In general, SP extraction procedures involve a preliminary treatment of seaweed tissue with an organic solvent to remove lipid and pigments as much as possible, followed by proteolytic digestion [18] and acid and/or alkaline extraction [19,20]. In connection with the emerging concept of “Green Chemistry”, ultrasound-assisted extraction has been applied to extract polysaccharides from plant tissues [21]; however, sonication has not yet been used to extract SP from seaweed.

Thus, the main objective of this study was to obtain sulfated polysaccharides using different conditions, such as neutral or alkaline extraction, sonication and/or proteolytic digestion, in order to identify the best condition to obtain SP from the cultivated and edible algae, *G.* *birdiae*. The use of sonication for the extraction of SP from seaweeds was utilized and tested here, as it has not previously been reported. In addition, the anticoagulant and antioxidant activities of these extracts were evaluated.

## 2. Results and Discussion

### 2.1. Extraction Conditions

In order to obtain sulfated polysaccharide-rich extracts from *G. birdiae*, different extraction conditions reported by several authors were used. GB1 extraction was performed as described by Maciel and colleagues [22], who obtained galactans from *G. birdiae* [22]. In previous studies, sonication has been used to optimize the yield of compound extracts obtained from plants [23,24], as ultrasound acts in a process named cavitation, which produces shock waves that affect plant cell wall integrity; this increases the extraction of several compounds, including less accessible polysaccharides, in a shorter time and at lower temperatures [21]. In the current study, sonication was used to improve GB1 condition, and this new extraction condition was named GB1s. In addition, a combination of sonication and alkaline solution was used to obtain GB2s, since it was reported that the presence of NaOH improves the polysaccharide extraction with sonication [25].

Enzymatic digestion has been used to extract sulfated polysaccharides from seaweed [1,18]. In the seaweed extracellular matrix, the proteins act as a kind of jail for the polysaccharides. As such, when the proteolitic enzyme breaks down the proteins from the extracellular matrix, the extraction of polysaccharides becomes possible. Thus, in order to assess whether the use of proteolitic extraction could increase the efficiency of sonication, enzymatic digestion was combined with GB1s and GB2s to obtain GB1sp (p, proteolysis) and GB2sp, respectively. Since the proteolytic enzymes do not work at pH levels less than 6.5, acidic conditions were not used for SP extraction.

### 2.2. Yield and Chemical Analysis of Extracts Obtained

As shown in Table 1, each of the five tested conditions showed different yields, ranging from 26 to 413 mg. Following the work of Maciel *et al.* [22], when water alone was used to extract SP (GB1) a low yield was obtained, and this was not improved when combined with sonication (GB1s). This result was unexpected, as several authors have cited the use of sonication in order to increase the extraction of polysaccharides in plants [21,23,26]. Wang and Zhang [25] showed that when using sonication in an alkaline system, the extraction of polysaccharides in corn cobs increases; thus, in the current study, when we also extracted SP from *G. birdiae* using sonication in the presence of water and NaOH (GB2s), an increased yield was observed in comparison to GB1 and GB1s.

Another condition used by many researchers to extract SP from seaweed is enhancing the degradation of proteins that are associated or have covalent bonds with the polysaccharides of the algae, allowing for solubilization of SP in water, a condition known as proteolysis [1,17]. As such, proteolysis was combined with sonication (GB1sp) in order to improve the yield of extraction in *G. birdiae*. As shown in Table 1, this combination improved the yield when compared to the others cited. This occurred probably because the tissue of the extracellular matrix became softer due to the ultrasound used, which helped the action of the proteolytic enzymes. In addition, the mixture of an alkaline solution, proteolysis and sonication (GB2sp) was used, and this condition showed the best yield, almost three-times greater than the second best (GB1sp).

**Table 1:** Chemical composition of extracts from *Gracilaria birdiae*. Gal, galactose; Glc, glucose; Ara, arabinose; Xy, xylose; Gluc A, glucuronic acid. * The value in parentheses refers to the ratio of the yield of each condition to the basic condition GB1 yields. s, sonication; p, proteolysis.

Extracts	Total Yield (mg)	Sugar/Sulfate	Protein (%)	Molar Ratio				
				Gal	Glc	Ara	Xyl	Gluc A
**GB1**	26 (1.0) *	8.5	0.0 ± 0.0	1.0	0.3	0.4	1.5	1.9
**GB1s**	28 (1.1) *	15.0	2.9 ± 0.5	1.0	0.5	0.3	1.5	0.9
**GB1sp**	152 (5.8) *	12.4	1.2 ± 0.4	1.0	0.3	0.3	1.3	2.2
**GB2s**	64 (2.5) *	11.0	0.4 ± 0.1	1.0	1.0	0.4	1.7	0.5
**GB2sp**	413 (15.9) *	5.1	0.4 ± 0.3	1.0	0.8	0.5	2.7	1.5

Total sugar, protein and sulfate are accounted for in Table 1. Upon comparing GB1 and GB1s, the presence of sonication led to the extraction of more proteins. However, the sugar/sulfate ratio increased, indicating that ultrasound promotes the extraction of other polysaccharides beyond that of SP, which allows one to conclude that sonication in water solution does not increase the extraction of SP. When using sonication in an alkaline solution (GB2s) or sonication with proteolytic digestion (GB1sp), it was observed that these conditions did not enable the extraction of more SP in comparison to GB1. On the other hand, when combining proteolysis, sonication and NaOH (GB2sp), it was observed that this condition extracted more material than the others. GB2sp also extracted more SP, since its sugar/sulfate ratio was the least of all of the conditions used. This happens because NaOH attacks the extracellular matrix eroded by ultrasound and cleaves the interaction between polysaccharides and phenolic compounds [21]; this, together with proteolitic enzyme action, enhances the release and solubilization of polysaccharides.

All samples showed galactose, glucose, arabinose and xylose as monosaccharaide components. However, these monosaccharaides were not found in the same proportions under all extraction conditions. GB1 presented a different monosaccharaide composition to that reported by Maciel *et al.* [22], who extracted a sulfated homogalactan using conditions to extract SP similar to what we used (GB1). This difference in monosaccharide composition might be due to the fact that the algae were collected in different years. Neutral and sulfated polysaccharides from the seaweed, *Saccharina longicruris*, showed different monosaccharaide composition when collected in 2005 and 2006 [27]. In addition, when SP extracts were obtained month by month from April to October from *Laminaria japonica*, their monosaccharide composition changed during these seven months [28].

GB2s and GB2sp extracted a larger amount of glucose when compared to the other conditions. Chanliaud *et al.*, 1995, reported that using an alkaline solution to extract heteroxylans from maize bran promotes the extraction of glucose, because of the degradation of cellulose and starch [29]. The same situation could justify the elevated presence of glucose in GB2sp and GB2s. Xylose was found in high quantities in GB2sp. Although sulfated galactans are the main sulfated polysaccharide found in red algae, some authors have reported the presence of sulfated polysaccharides rich in xylose in red algae [30]. To date, there have been no reports showing the different types of sulfated polysaccharides that are synthesized by *G. birdiae*, so this high amount of xylose in GB2sp may lead one to assume that *G. birdiae* also synthesizes sulfated polysaccharides rich in xylose. Further studies are needed to confirm this hypothesis. Glucuronic acid was found in low proportions in GB1s and GB2s, conditions that used sonication; however, GB1sp a GB2sp also used sonication and showed an elevated amount of glucuronic acid. Future studies should be undertaken to understand the effect of sonication on the extraction of glucuronic acid-rich polysaccharides from *G. birdiae*.

### 2.3. Agarose Gel Electrophoresis

**Figure 1:**
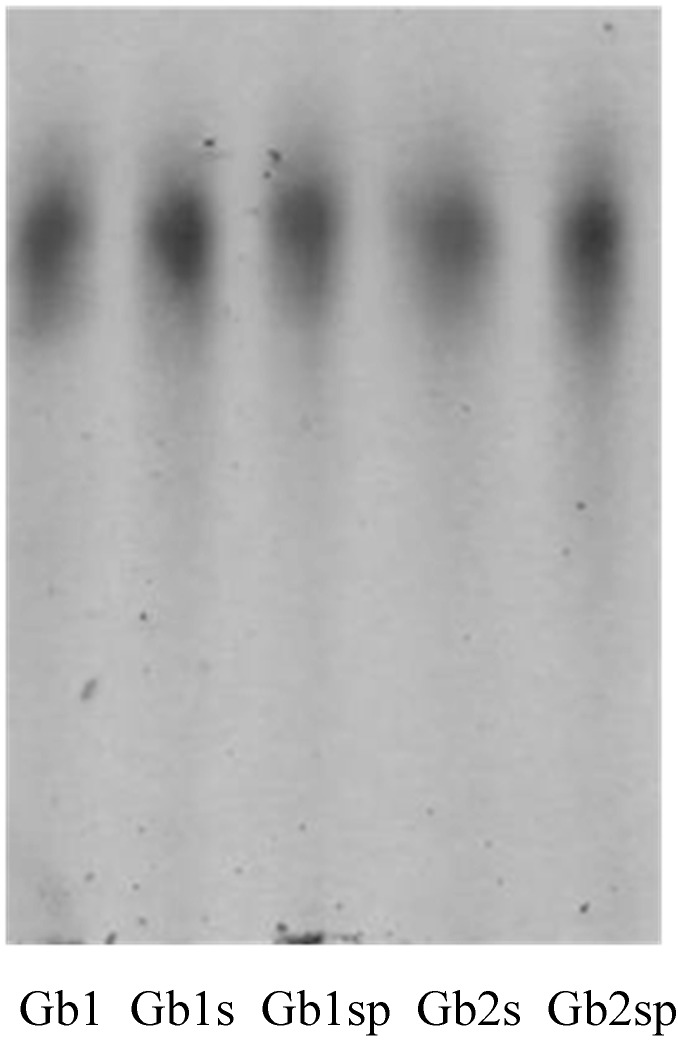
Agarose gel electrophoresis of the extracts from *Gracilaria birdiae*. About 5-µL aliquots (50 µg) of *Gracilaria* polysaccharides were applied in agarose gel (107.5 cm, 0.2 cm thick) prepared in 0.05 M 1,3-diaminopropane-acetate buffer (pH 9.0) and subjected to electrophoresis at 110 V/cm for 60 minutes. The gels were then maintained in 0.1% cetyltrimethylammonium bromide for two hours and dried, and the polysaccharides were stained with 0.1% toluidine blue in a solution containing 50% ethanol and 1% acid acetic in water for 15 min. The gels were then destained with the same solution lacking toluidine blue.

The electrophoretic mobility of sulfated polysaccharides on agarose gel, using a diaminopropane/acetate buffer, is shown in Figure 1.

The staining pattern of polysaccharides on the agarose gel electrophoresis with toluidine blue revealed that all extraction conditions obtained sulfated polysaccharides. In addition, it was observed that only one band corresponding to PSs showed the same electrophoretic mobility in all extraction conditions. Due to the property of 1,3-diaminopropane/acetate buffer, it was possible to individualize the sulfated polysaccharides. Diamine is able to interact with the *Gracilaria* polysaccharides through their sulfated groups, as was previously observed for sulfated glycosaminoglycans [31]. This interaction is more dependent on the spacing of vicinal charges than the absolute charge of the compounds [31]. Thus, polysaccharides that have the same charge/mass ratio and structural conformation have similar electrophoretic mobilities in a diaminopropane/acetate buffer.

Due to the different monosaccharide composition shown by each extraction condition, one may deduce that the *G. birdiae* synthesized small amounts of other polysaccharides that were not visualized after staining with toluidine blue.

### 2.4. Infrared Spectra

Infrared spectroscopy has been shown to be a powerful tool to show similarities between the compounds. Table 2 shows the main bands observed in the IR spectra of the sulfated polysaccharides from *G. birdiae*.

The characteristic absorptions of sulfate were identified in the FTIR spectra of sulfated polysaccharides: bands around 1225–1254 cm^−1^ for an asymmetric S=O stretching vibration [32] and bands around 1040–1065 cm^−1^ for a symmetric C-O vibration associated with a C-O-SO_3_ group [33]. The peaks at 845–850 were caused by the bending vibration of C-O-S [34].

In addition, all fractions showed signals at 3423–3443 cm^−1^ and around 2920 cm^−1^, which were from the stretching vibration of O–H and C–H, respectively [35,36]. The bands around 1638–1654 cm^−1^ were due to the carboxyl group of uronic acid [37].

**Table 2:** IR spectrum data of sulfated polysaccharides from the red seaweed, *G. birdiae*.

Sulfated Polysaccharides	IR (cm^−1^)
**GB1**	3441, 2921, 1647, 1238, 1059, 847
**GB1s**	3442, 2917, 1650, 1234, 1040, 850
**GB2s**	3444, 2910, 1643, 1225, 1038, 845
**GB1sp**	3435, 2908, 1659, 1254, 1061, 847
**GB2sp**	3460, 2922, 1662, 1238, 1065, 849

### 2.5. Polyacrylamide Electrophoresis

The polyacrylamide analysis showed that polysaccharides extracted using different conditions had a molecular weight of ~45 kDa. An exception was observed for the one extracted with the GB2s condition; the polysaccharides extracted using this condition showed a low molecular weight of ~20 kDa. Several authors have shown that when ultrasound is used at different times to extract polysaccharides, the molecular weight of these molecules decreases if the time of exposure to ultrasound increases [38]. However, GB1s, GB1sp and GB2sp also used ultrasound, and the molecular weight of their polysaccharides did not decrease in comparison to the GB1 polysaccharide. Thus, one may conclude that the NaOH used in the GB2s condition decreased the molecular weight of the SP in GB2s or extracted only low molecular weight SP from *G. birdiae*.

### 2.6. Anticoagulant Activity

**Figure 2:**
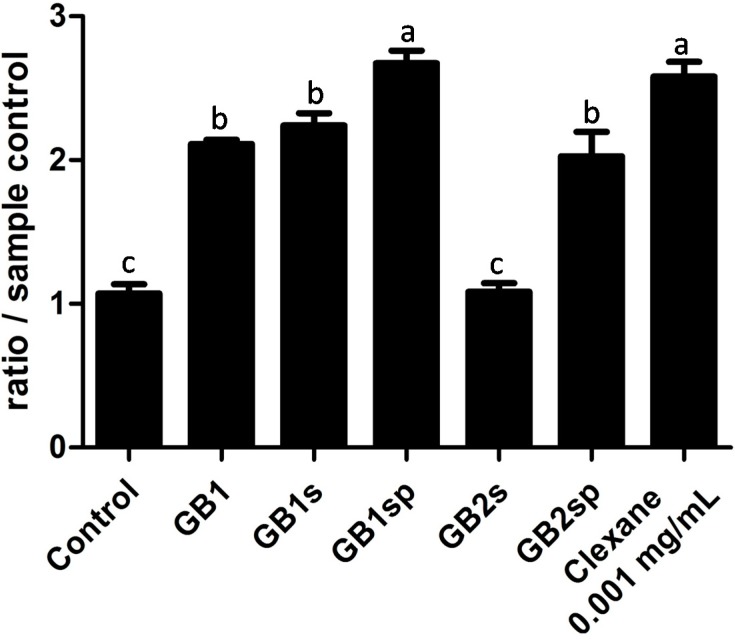
Anticoagulant activity in the activated partial thromboplastin time (aPTT) test of extracts from *Gracilaria birdiae.* Each value is the mean ± standard deviation of three determinations: a, b, c, different letters refer to a significant difference (*p* < 0.05) between each extraction. Clexane^®^ was used as a positive control.

Polysaccharides from *G. birdiae* presented anticoagulant activity, except GB2s, as shown in Figure 2. GB1sp showed the best activity, followed by GB1s, GB1 and GB2sp. Anticoagulant activity is more common in fucans from brown seaweed, such as *Dictyopteris delicatula* [39], and galactans from green seaweed, like *Caulerpa cupressoides* [40]. Nonetheless, the literature mentions several galactans from red seaweed with anticoagulant activity [1]. A decrease in the MW of (1→3) β-glucans (curdlans) was accompanied by a decrease in their anticoagulant activity in three standard tests (activated partial thromboplastin time (aPTT), TT and Heptest). Anticoagulant curdlans have a molecular weight higher than 30 kDa [41]. Sulfated galactans from the red seaweed *B. occidentalis* with a molecular weight of 15–45 kDa bound to antithrombin, but were not able to form complexes with thrombin and plasma inhibitors. Thus, they did not show anticoagulant activity. The last process requires a MW of over 45 kDa [42]. This study, therefore, concludes that the SP from GP2s did not show anticoagulant activity, due to its low molecular weight.

### 2.7. Antioxidant Activity

**Figure 3:**
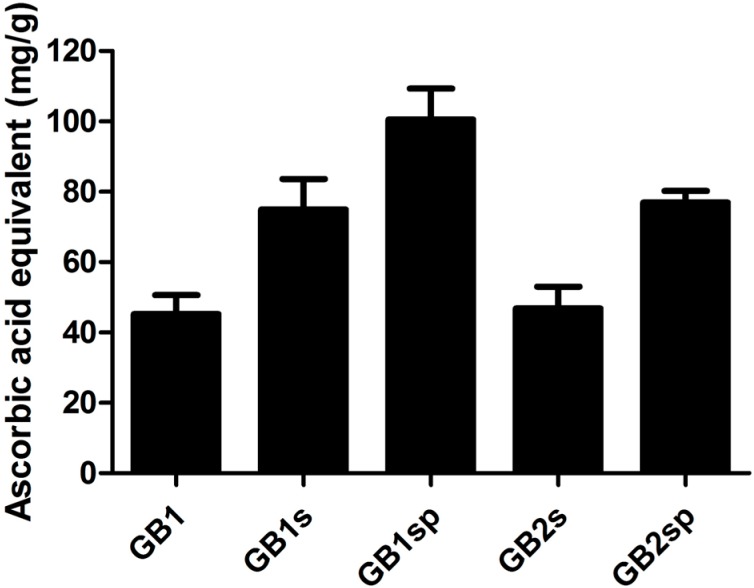
Total antioxidant capacity of extracts from *Gracilaria birdiae.* The results are expressed as ascorbic acid equivalents. Each value is the mean ± standard deviation of two determinations: a, b, different letters refer to significant difference (*p* < 0.05) between each extraction.

Several reports have shown that many different *in vitro* antioxidant methods are being used to evaluate the activity of biomolecules [43]. In all antioxidant tests used in this article, the SP from *G. birdiae* showed activity only in the total antioxidant capacity, ranging from 41.6 mg/g to 75.9 mg/g of ascorbic acid equivalent (Figure 3). The presence of antioxidant activity in this assay indicates that SPs interact with the systems sending electrons in order to minimize the free radical attack. The highest activity found was from GB1sp, GB1s and GB2sp.

Total antioxidant capacity is commonly found in SP from algae. However, each seaweed sensitizes SP with different antioxidant activities. The results observed here showed SP with high activity. The literature has reported SP with lower antioxidant activity, such as SP from *Turbinaria conoides* and *Padina tetrastomatica*, which showed total antioxidant capacity of 9.65 mg/g and 9.79 mg/g, respectively [44], and heterofucans from the seaweed *Dictyopteris delicatula* and *Canistrocarpus cervicornis*, which presented total antioxidant capacity ranging from 18.8 mg/g to 22.8 mg/g [39,45].

Other antioxidant assays were evaluated: hydroxyl radical scavenging, superoxide radical scavenging, ferrous ion chelating and power reducing have no significant antioxidant activity until a concentration of 1 mg/mL of SP, as shown in Table 3.

**Table 3:** Hydroxyl radical scavenging, superoxide radical scavenging, ferrous ion chelating and power reducing of sulfated polysaccharides extractions from the red seaweed, *Gracilaria birdiae*.

Extracts (1 mg/mL)	Hydroxyl Radical Scavenging	Superoxide Radical Scavenging	Ferrous Ion Chelating	Power Reducing
**GB1**	−0.02 ± 0.03 ^a^	0.00 ± 0.23 ^a^	0.19 ± 0.24 ^a^	Nd
**GB1p**	−0.09 ± 0.12 ^a^	0.00 ± 0.51 ^a^	0.21 ± 0.12 ^a^	Nd
**GB1s**	−0.10 ± 0.33 ^a^	0.00 ± 0.02 ^a^	0.08 ± 0.27 ^a^	Nd
**GB1sp**	0.01 ± 0.09 ^a^	0.00 ± 0.33 ^a^	0.05 ± 0.10 ^a^	Nd
**GB2s**	−0.27 ± 0.45 ^a^	0.00 ± 0.12 ^a^	0.05 ± 0.17 ^a^	Nd
**GB2sp**	−0.11 ± 0.09 ^a^	0.00 ± 0.31 ^a^	0.09 ± 0.11 ^a^	Nd

Each value is the mean ± SD, ^a^ Letter indicates same significant (*p* < 0.05) between each extraction; Nd, not detected.

## 3. Experimental Section

### 3.1. Materials

Potassium ferricyanide, ferrous sulfate II and sulfuric acid were obtained from Merck (Darmstadt, Germany). Human blood was collected in 0.82% citrate of sodium and centrifuged to separate the plasma; aliquots of 10 mL of plasma were taken and frozen at −20 °C in sterile bottles. Sodium bicarbonate, non-essential amino acids, sodium pyruvate and phosphate buffered saline (PBS) were purchased from Invitrogen Corporation (Burlington, VT, USA). All other solvents and chemicals were of analytical grade.

### 3.2. Raw Material

The red seaweed, *Gracilaria birdiae*, was collected in Rio do Fogo Beach (Rio Grande do Norte, Brazil, 5°16'16.61"S/35°22'54.29"W) by fishermen from the community; the seaweed was exposed to the morning Sun in order to be dried. The dried seaweed was taken to the laboratory and cleaned to eliminate residue and epiphytes. This material was then powdered, and 200 mL of ethanol were applied to the material overnight 5-times to reduce pigments, in line with the protocol described by Leite *et al.*, 1998 [46]. The supernatant was eliminated, and the red seaweed was dried at 50 °C under ventilation. The dilapidated dried material was packed in polyethylene bags and stored at room temperature in the dark.

### 3.3. Extraction of Sulfated Polysaccharide

Production was based on the different conditions to solubilize sulfated polysaccharides from the red seaweed, using sonication, proteolytic digestion and different solvents. Five grams of the raw material of *Gracilaria birdiae* were put into 5 separate beakers. Each beaker was subjected to different treatment to extract sulfated polysaccharides; the condition of each methodology is described in Table 4. After the extraction, the sulfated polysaccharide-rich extracts were dried in a vacuum bomb and then were ready for biological assays.

**Table 4:** Conditions to extract sulfated polysaccharides from *Gracilaria birdiae*.

Extract	Solution	Sonication (30 min/60 °C/60 W)	Enzymatic Digestion (60 °C, 12 h, pH 8.0)
GB1	Water 22 °C	-	-
GB1s	Water 22 °C	+	-
GB2s	0.1 M NaOH 22 °C	+	-
GB1sp	Water 22 °C	+	+
GB2sp	0.1 M NaOH 22 °C	+	+

### 3.4. Chemical Analysis and Monosaccharide Composition

Total sugars were estimated by the phenol-H_2_SO_4_ reaction [47] using D-galactose as the standard. Sulfate content was determined according to the gelatin-barium method [48], using sodium sulfate (1 mg/mL) as the standard, followed by acid hydrolysis of the polysaccharides (4 M HCl, 100 °C, 6 h). Protein content was measured using Spector’s method [49].

The polysaccharides were hydrolyzed with 0.5, 1, 2 and 4 M, respectively, for various lengths of time (0.5, 1, 2 and 4 h), at 100 °C. Reducing sugars were determined as described earlier [50]. After acid hydrolysis, sugar composition was determined by a LaChrom Elite^®^ HPLC system from VWR-Hitachi with a refractive index detector (RI detector model L-2490). A LichroCART^®^ 250-4 column (250 mm × 40 mm) packed with Lichrospher^®^ 100 NH_2_ (5 µm) was coupled with the system. The sample mass used was 0.2 mg, and the analysis time was 25 min. The following sugars were analyzed as references: arabinose, fructose, fucose, galactose, glucose, glucosamine, glucuronic acid, mannose and xylose.

### 3.5. Agarose Electrophoresis

This method consists of a separation of molecules according to their interaction with 0.05 M PDA (diamine-propane acetate) buffer and their exposing negative charges. The agarose gel was prepared in 0.6% with 0.05 M PDA buffer and molded in a blade glass (7.5 cm × 7.5 cm × 15 mm or 5.0 cm × 7.5 cm × 15 mm). Five microliters of each sample containing the extracts were applied in spots present in the gel and submitted to electrophoresis with 90 V in a refrigerated system (4 °C). On conclusion of the electrophoresis, the agarose blade was soaked in 0.1% CTV (cetavlon) for 2 h at room temperature. After 2 h, the blade was exposed to hot air in order to dry it. As soon as the blade was dry, toluidine blue reagent was applied in the blade, being stirred every 15 min. Finally, the excess of dye was eliminated with acetic acid/ethanol/water, and the blade was analyzed [31].

### 3.6. Infrared Spectra

Sulfated polysaccharides (5 mg) of each extraction were mixed thoroughly with dry potassium bromide. A pellet was prepared, and the infrared spectra between 500 and 4000 cm^−1^ were measured on a Thermo-Nicolet Nexus 470 ESP FTIR spectrometer. Thirty-two scans at a resolution of 4 cm^−1^ were averaged and referenced against air.

### 3.7. Polyacrylamide Electrophoresis

Following the protocol of Dietrich and Nader (1974), TBE buffer and polyacrylamide gel were used to separate molecules by obstacles [51]. When the electrophoresis starts, this gel separates molecules according to their structure and weight. In this study, polyacrylamide gel was prepared in a blade support and put into an electrophoretic vat. Before the electrophoresis started, each extract of the red seaweed was applied in spots on this gel. In conclusion, the gel was dyed with 0.1% toluidine blue, and the excess was eliminated with acetic acid/ethanol/water.

### 3.8. Anticoagulant Activity

Prothrombin time (PT) and activated partial thromboplastin time (aPTT) coagulation assays were performed with a coagulometer, as described earlier [50], and measured using normal citrate-treated human plasma. All assays were performed in duplicate and repeated at least three times on different days (*n* = 6). The results were expressed as aPTT ratio, which was determined as follows: aPTT control time/aPTT sample time.

### 3.9. Antioxidant Activity

Five assays were performed to analyze the antioxidant activity of the sulfated polysaccharides obtained: total antioxidant capacity, hydroxyl radical scavenging, superoxide radical scavenging, ferric chelating and power reducing, as previously described [52].

#### 3.9.1. Determination of Total Antioxidant Capacity

This assay is based on the reduction of Mo (VI) to Mo (V) by sulfated polysaccharides and subsequent formation of a green phosphate/Mo(V) complex at acidic pH levels. Tubes containing sulfated polysaccharides and reagent solution (0.6 M sulfuric acid, 28 mM sodium phosphate and 4 mM ammonium molybdate) were incubated at 95 °C for 90 min. After the mixture had cooled to room temperature, the absorbance of each solution was measured at 695 nm against a blank. Total antioxidant capacity was expressed as ascorbic acid equivalent.

#### 3.9.2. Hydroxyl Radical Scavenging Activity Assay

The scavenging activity of seaweed polysaccharides against the hydroxyl radical was investigated using Fenton’s reaction (Fe^2+^ + H_2_O_2_→Fe^3+^ + OH^−^ + OH˙). These results were expressed as the inhibition rate. Hydroxyl radicals were generated using 3 mL sodium phosphate buffer (150 mM, pH 7.4), which contained 10 mM FeSO_4_·7H_2_O, 10 mM EDTA, 2 mM sodium salicylate, 30% H_2_O_2_ (200 mL) and varying polysaccharide concentrations. In the control, sodium phosphate buffer replaced H_2_O_2_. The solutions were incubated at 37 °C for 1 h, and the presence of the hydroxyl radical was detected by monitoring absorbance at 510 nm. Gallic acid was used as the positive control.

#### 3.9.3. Superoxide Radical Scavenging Activity Assay

This assay was based on the capacity of sulfated polysaccharides to inhibit the photochemical reduction of nitroblue tetrazolium (NBT) in the riboflavin-light-NBT system. Each 3 mL of the reaction mixture contained 50 mM phosphate buffer (pH 7.8), 13 mM methionine, 2 mM riboflavin, 100 mM EDTA, NBT (75 mM) and 1 mL sample solution. After the production of blue formazan, the increase in absorbance at 560 nm after 10 min illumination from a fluorescent lamp was determined. The entire reaction assembly was enclosed in a box lined with aluminum foil. Identical tubes with the reaction mixture were kept in the dark and served as blanks. Gallic acid was used as the positive control.

#### 3.9.4. Ferrous Ion (Fe(II)) Chelating Activity

The ferrous ion chelating ability of samples was investigated using the following methodology: sulfated polysaccharides at different concentrations were applied with the reaction mixture, which contained FeCl_2_ (0.05 mL, 2 mM) and ferrozine (0.2 mL, 5 mM). The mixture was shaken and incubated for 10 min at room temperature, and the absorbance of the mixture was measured at 562 nm against a blank. EDTA was used as the positive control.

#### 3.9.5. Power Reducing

The reducing power of the samples was quantified as described previously. Briefly, 4 mL of reaction mixture, containing different sample concentrations in phosphate buffer (0.2 M, pH 6.6), was incubated with potassium ferricyanide (1% w/v) at 50 °C for 20 min. The reaction was stopped by TCA solution (10% w/v). The solution was then mixed with distilled water and ferric chloride (0.1% w/v) solution, and the absorbance was measured at 700 nm. The result was expressed as a percentage of the activity shown by 0.2 mg/mL of vitamin C.

### 3.10. Statistical Analysis

All data are expressed as the mean ± standard deviation. Statistical analysis was done by one-way ANOVA using the SIGMAStat 2.01 software. Student–Newman–Keuls post-tests were performed for multiple group comparison. In all cases, statistical significance was set at *p* < 0.05.

## 4. Conclusions

Five different conditions were utilized to extract sulfated polysaccharides from the red seaweed, *Gracilaria birdiae*: GB1, GB1s, GB1sp, GB2s and GB2sp. Infrared and agarose electrophoreses showed that the same SP was extracted in all conditions. Only GB2s did not show anticoagulant activity; all of the other conditions showed anticoagulant activity in aPTT tests. However, no samples had anticoagulant activity in the PT test. It was observed that all samples showed a great total antioxidant capacity in comparison to the polysaccharide-rich extract from other seaweeds. The data showed that NaOH/sonication/proteolysis was the best condition to extract anticoagulant and antioxidant SPs from *Gracilaria birdiae*. In addition, SPs from *Gracilaria birdiae* was identified as a potential antioxidant and anticoagulant drug.

## References

[1] Costa L.S., Fidelis G.P., Cordeiro S.L., Oliveira R.M., Sabry D.A., Câmara R.B.G., Nobre L.T.D.B., Costa M.S.S.P., Almeida-Lima J., Farias E.H.C. (2010). Biological activities of sulfated polysaccharides from tropical seaweeds. Biomed. Pharmacother..

[2] Albuquerque I.R., Queiroz K.C., Alves L.G., Santos E.A., Leite E.L., Rocha H.A.O. (2004). Heterofucans from *Dictyota menstrualis* have anticoagulant activity. Braz. J. Med. Biol. Res..

[3] Nader H.B., Lopes C.C., Rocha H.A.O., Santos E.A., Dietrich C.P. (2004). Heparins and heparinoids: Occurrence, structure and mechanism of antithrombotic and hemorrhagic activities. Curr. Pharm. Des..

[4] Ciancia M., Quintana I., Cerezo A.S. (2010). Overview of anticoagulant activity of sulfated polysaccharides from seaweeds in relation to their structures, focusing on those of green seaweeds. Curr. Med. Chem..

[5] Rodrigues J.A.G., Queiroz I.N.L., Bessa E.F., Coura C.O., Amorim R.C.N., Benevides N.M.B. (2011). Anticoagulant activity of sulfated polysaccharides fractions from an aqueous extract obtained from the red seaweed *Halymenia floresia* (Clemente) C. Agardh. Acta Sci. Technol..

[6] Queiroz K.C.S., Assis C.F., Medeiros V.P., Rocha H.A.O., Aoyama H., Ferreira C.V., Leite E.L. (2006). Cytotoxicity effect of algal polysaccharides on HL60 cells. Biochemistry (Mosc).

[7] Plastino E.M., Ursi S., Fujii M.T. (2004). Color inheritance, pigment characterization, and growth of a rare light green strain of *Gracilaria birdiae* (Gracilariales, Rhodophyta). Phycol. Res..

[8] Bezerra A.F., Marinho-Soriano E. (2010). Cultivation of the red seaweed *Gracilaria birdiae* (Gracilariales, Rhodophyta) in tropical waters of northeast Brazil. Biomass Bioenergy.

[9] Marinho-Soriano E., Moreira W.S.C., Carneiro M.A.A. (2006). Some aspects of the growth of *Gracilaria birdiae* (Gracilariales, Rhodophyta) in an Estuary in Northeast Brazil. Aquac. Int..

[10] Silva R.O., Santana A.P., Carvalho N.S., Bezerra T.S., Oliveira C.B., Damasceno S.R., Chaves L.S., Freitas A.L., Soares P.M., Souza M.H. (2012). A sulfated-polysaccharide fraction from seaweed *Gracilaria birdiae* prevents naproxen-induced gastrointestinal damage in rats. Mar. Drugs.

[11] Brito T.V., Neto J.P., Prudêncio R.S., Batista J.A., Júnior J.S., Silva R.O., Franco A.X., Aragão K.S., Soares P.M., Souza M.H. (2014). Sulfated-polysaccharide fraction extracted from red algae *Gracilaria birdiae* ameliorates trinitrobenzenesulfonic acid-induced colitis in rats. J. Pharm. Pharmacol..

[12] Souza B.W.S., Cerqueira M.A., Bourbon A.I., Pinheiro A.C., Martins J.T., Teixeira J.A., Coimbra M.A., Vicente A.A. (2012). Chemical characterization and antioxidant activity of sulfated polysaccharide from the red seaweed *Gracilaria birdiae*. Food Hydrocoll..

[13] Vanderlei E.S.O., Araujo I.W.F., Quindere A.L.G., Fontes B.P., Eloy Y.R.G., Rodrigues J.A.G., Silva A.A.R., Chaves H.V., Jorge R.J.B., Menezes D.B. (2011). The involvement of the HO-1 pathway in the anti-inflammatory action of a sulfated polysaccharide isolated from the red seaweed Gracilaria birdiae. Inflamm. Res..

[14] Pomin V.H., Mourão P.A. (2008). Structure, biology, evolution, and medical importance of sulfated fucans and galactans. Glycobiology.

[15] Grauffel V., Kloareg B., Mabeu S., Durand P., Jozefonvicz J. (1989). New natural polysaccharides with potent antithrombotic activity: Fucans from brown algae. Biomaterials.

[16] Chevolot L., Foucault A., Chaubet F., Kervarec N., Sinquin C., Fisher A., Boisson-Vidal C. (1999). Further data on the structure of brown seaweed fucans: Relationships with anticoagulant activity. Carbohydr. Res..

[17] Ale M.T., Mikkelsen J.D., Meyer A.S. (2011). Review of structure-function relations and extraction methods for fucose-containing sulfated polysaccharides from brown seaweeds. Mar. Drugs.

[18] Rocha H.A.O., Bezerra L.C., Albuquerque I.R.L., Costa L.S., Guerra C.M., Abreu L.D., Nader H.B., Leite E.L. (2005). A xylogalactofucan from the brown seaweed S*patoglossum schroederi* stimulates the synthesis of an antithrombotic heparan sulfate from endothelial. Planta Med..

[19] Leiro J.M., Castro R., Arranz J.A., Lamas J. (2007). Immunomodulating activities of acidic sulphated polysaccharides obtained from the seaweed *Ulva rigida* C. Agardh. Int. Immunopharmacol..

[20] Estevez J.M., Ciancia M., Cerezo A.S. (2008). The system of sulfated galactans from the red seaweed *Gymnogongrus torulosus* (Phyllophoraceae, Rhodophyta): Location and structural analysis. Carbohydr. Polym..

[21] Hromádková Z., Košt’álová Z., Ebringerová A. (2008). Comparison of conventional and ultrasound-assisted extraction of phenolics-rich heteroxylans from wheat bran. Ultrason. Sonochem..

[22] Maciel J.S., Chaves L.S., Souza B.W.S., Teixeira D.I.A., Freitas A.L.P., Feitosa J.P.A., de Paula R.C.M. (2008). Structural characterization of cold extracted fraction of soluble sulfated polysaccharide from red seaweed *Gracilaria birdiae*. Carbohydr. Polym..

[23] Melo-Silveira R.F., Fidelis G.P., Costa M.S.S.P., Telles C.B., Dantas-Santos N., Oliveira E.S., Ribeiro V.B., Barth A.L., Macedo A.J., Leite E.L. (2012). *In vitro* antioxidant, anticoagulant and antimicrobial activity and in inhibition of cancer cell proliferation by xylan extracted from corn cobs. Int. J. Mol. Sci..

[24] Lai F.R., Wen Q.B.A., Li L., Wu H., Li X.F. (2010). Antioxidant activities of water-soluble polysaccharide extracted from mung bean (*Vigna radiata* L.) hull with ultrasonic assisted treatment. Carbohydr. Polym..

[25] Wang Y., Zhang J. (2006). A novel hybrid process, enhanced by ultrasonication, for xylan extraction from corncobs and hydrolysis of xylan to xylose by xylanase. J. Food Eng..

[26] Ebringerová A., Hromfidková Z., Hríbalová V., Mason T.J. (1997). Effect of ultrasound on the immunogenic corn cob xylan. Ultrason. Sonochem..

[27] Rioux L.E., Turgeon S.L., Beaulieu M. (2009). Effect of season on the composition of bioactive polysaccharides from the brown seaweed S*accharina longicruris*. Phytochemistry.

[28] Honya M., Mori H., Anzai M., Araki Y., Nisizawa K. (1999). Monthly changes in the content of fucans, their constituent sugars and sulphate in cultured *Laminaria japonica*. Hydrobiologia.

[29] Chanliaud E., Saulnier L., Thibault J.-F. (1995). Alkaline extraction and characterisation of heteroxylans from maize bran. J. Cereal Sci..

[30] Mandal P., Pujol C.A., Carlucci M.J., Chattopadhyay K., Damonte E.B., Ray B. (2008). Anti-herpetic activity of a sulfated xylomannan from *Scinaia hatei*. Phytochemistry.

[31] Dietrich C.P., Dietrich S.M.C. (1976). Electrophoretic behavior of acidic mucopolysaccharides by agarose gel electrophoresis. J. Chromatogr..

[32] Zhang Z., Zhang Q., Wang J., Zhang H., Niu X., Li P. (2009). Preparation of the different derivatives of the low-molecular-weight porphyran from *Porphyra haitanensis* and their antioxidant activities *in vitro*. Int. J. Biol. Macromol..

[33] Zhang Z., Wang F., Wang X., Liu X., Hou Y., Zhang Q. (2010). Extraction of the polysaccharides from five algae and their potential antioxidant activity *in vitro*. Carbohydr. Polym..

[34] Zhang H.J., Mao W.J., Fang F., Li H.Y., Sun H.H., Chen Y., Qi X.H. (2008). Chemical characteristics and anticoagulant activities of a sulfated polysaccharide and its fragments from *Monostroma latissimum*. Carbohydr. Polym..

[35] Sekkal M., Legrand P. (1993). A spectroscopic investigation of the carrageenans and agar in the 1500–100 cm^−1^ spectral range. Spectrochim. Acta Part A Mol. Biomol. Spectroc..

[36] Lahaye M., Rochas C., Yaphe W. (1986). A new procedure for determining the heterogeneity of agar polymer in the cell walls of *Gracilaria spp* (Gracilariaceae, Rhodophyta). Can. J. Bot..

[37] Silva T.M., Alves L.G., de Queiroz K.C., Santos M.G., Marques C.T., Chavante S.F., Rocha H.A., Leite E.L. (2005). Partial characterization and anticoagulant activity of a heterofucan from the brown seaweed *Padina gymnospora*. Braz. J. Med. Biol. Res..

[38] Zhou C., Wang Y., Ma H., He R. (2008). Effect of Ultrasonic Degradation on *In Vitro* Antioxidant Activity of Polysaccharides from *Porphyra yezoensis* (Rhodophyta). Food Sci. Technol. Int..

[39] Magalhaes K.D., Costa L.S., Fidelis G.P., Oliveira R.M., Nobre L.T.D.B., Dantas-Santos N., Câmara R.B.G., Albuquerque I.R.L., Cordeiro S.L., Sabry D.A. (2011). Anticoagulant, Antioxidant and Antitumor Activities of Heterofucans from the Seaweed *Dictyopteris delicatula*. Int. J. Mol. Sci..

[40] Costa M.S.SP., Costa L.S., Cordeiro S.L., Almeida-Lima J., Dantas-Santos N., Magalhães K.D., Sabry D.A., Albuquerque I.R.L., Pereira M.R., Leita E.L. (2012). Evaluating the possible anticoagulant and antioxidant effects of sulfated polysaccharides from tropical seaweed *Caulerpa cupressoides* var. *flabellata*. J. Appl. Phycol..

[41] Mestechkina N.M., Shcherbukhin V.D. (2010). Sulfated Polysaccharides and Their Anticoagulant Activity: A Review. Appl. Biochem. Microbiol..

[42] Pereira M.G., Benevides N.M.B., Melo M.R.S., Valente A.P., Melo F.R., Mourao P.A.S. (2005). Structure and anticoagulant activity of a sulfated galactan from the red alga, *Gelidium crinale*. Is there a specific structural requirement for the anticoagulant action?. Carbohydr. Res..

[43] Krishnaiah D., Sarbatly R., Nithyanandam R. (2011). A review of the antioxidant potential of medicinal plant species. Food Bioprod. Process.

[44] Chandini S.K., Ganesan P., Bhaskar N. (2008). *In vitro* antioxidant activities of three selected brown seaweeds of India. Food Chem..

[45] Camara R.B.G., Costa L.S., Fidelis G.P., Nobre L.T.D.B., Dantas-Santos N., Cordeiro S.L., Costa M.S.S.P., Alves L.G., Rocha H.A.O. (2011). Heterofucans from the Brown Seaweed *Canistrocarpus cervicornis* with Anticoagulant and Antioxidant Activities. Mar. Drugs.

[46] Leite E.L., Medeiros M.G.L., Rocha H.A.O., Farias G.G.M., Silva L.F., Chavante S.F., Dietrich C.P., Nader H.B. (1998). Structure of a new fucan from the algae *Spatoglossum Schröederi*. Plant Sci..

[47] Dubois M., Gilles K.A., Hamilton J.K., Rebers P.A., Smith F. (1956). Colorimetric method for determination of sugars and related substances. Anal. Chem..

[48] Dodgson K.S., Price R.G. (1962). A note on the determination of the ester sulphate content of sulphated polysaccharides. Biochem. J..

[49] Spector J. (1978). Refinement of the coomassie blue method of protein quantification. A simple and linear spectrophotometric assay of 0.5 to 50 μg of protein. Anal. Biochem..

[50] Barroso E.M., Costa L.S., Medeiros V.P., Cordeiro S.L., Costa M.S.S.P., Franco C.R.C., Nader H.B., Leite E.L., Rocha H.A.O. (2008). A non-anticoagulant heterofucan has antithrombotic activity *in vivo*. Planta Med..

[51] Dietrich C.P., Nader H.B. (1974). Fractionation and properties of four heparitin sulfates from beef lung tissue: Isolation and characterization of a homogeneous species of heparitin sulfate. Biochem. Biophys. Acta.

[52] Costa L.S., Fidelis G.P., Telles C.B., Dantas-Santos N., Camara R.B.G., Cordeiro S.L., Costa M.S.S.P., Lima J.A., Silveira R.F.M., Oliveira R.M. (2011). Antioxidant and Antiproliferative Activities of Heterofucans from the Seaweed *Sargassum filipendula*. Mar. Drugs.

